# Evaluation of New Generation Sequencing (NGS)-Based Somatic Gene Variations and Real-Time Polymerase Chain Reaction (PCR)-Based Gene Fusions in Elderly and Young Acute Leukemia Patients: A Retrospective View

**DOI:** 10.3390/jpm14020140

**Published:** 2024-01-26

**Authors:** İbrahim Halil Erdoğdu, Seda Örenay-Boyacıoğlu, Olcay Boyacıoğlu, Nesibe Kahraman-Çetin, Füruzan Kacar-Döger, İrfan Yavaşoğlu, Ali Zahit Bolaman

**Affiliations:** 1 Department of Molecular Pathology, Faculty of Medicine, Aydin Adnan Menderes University, Aydin 09010, Türkiye; ibrahim.halil.erdogdu@adu.edu.tr (İ.H.E.); nesibe.cetin@adu.edu.tr (N.K.-Ç.); fdoger@adu.edu.tr (F.K.-D.); 2 Department of Medical Genetics, Faculty of Medicine, Aydin Adnan Menderes University, Aydin 09010, Türkiye; 3 Faculty of Engineering, Aydin Adnan Menderes University, Aydin 09010, Türkiye; oboyaci@adu.edu.tr; 4 Department of Hematology, Faculty of Medicine, Aydin Adnan Menderes University, Aydin 09010, Türkiye; iyavasoglu@adu.edu.tr (İ.Y.); zbolaman@adu.edu.tr (A.Z.B.)

**Keywords:** geriatrics, acute leukemia, gene variations, gene fusions, NGS

## Abstract

Malignant diseases occurring in elderly patients follow a different course from younger patients and show different genetic structures. Therefore, in this retrospective study, the somatic gene variant profile and fusion gene profiles of elderly and young acute leukemia patients were determined to draw attention to the existing genetic difference, and the results were compared. In this study, the records of 204 acute leukemia patients aged 18+ who were referred to the Molecular Pathology Laboratory from the Hematology Clinic between 2018 and 2022 were reviewed retrospectively. Fusion gene detection in patients was performed with the HemaVision^®^-28Q Panel. The NGS Myeloid Neoplasms Panel was conducted using the MiniSEQ NGS platform according to the manufacturer’s protocol. When all cases are evaluated together, the most frequently diagnosed acute leukemia is acute myeloid leukemia (85.8%). Both groups had a similar fusion gene profile; however, the fusion burden was higher in the elderly group. When the groups were evaluated in terms of somatic gene variations, there were differences between the groups, and the variation load was higher in the elderly group. Considering the different somatic gene variation profiles, it is understood that the genetic structure of tumor cells is different in elderly patients compared to young cases.

## 1. Introduction

Malignancies occur at any age, but the incidence of cancer increases with age and is more common in elderly patients. Cancer is the leading cause of death among men and women aged 60–79 years; 60% of newly diagnosed cancers are seen in patients aged 65 and over, and 70% of cancer deaths occur in this age group [1,2]. Due to demographic changes (population aging and increasing life expectancy), the proportion of elderly cancer patients is expected to increase further in the coming decades. It is estimated that 70% of all cancers will afflict patients aged 65 and over by 2030 [2].

According to the World Health Organization (WHO), hematological malignancies constitute 6.5% of all cancers and are responsible for 7.2% of cancer-related deaths [3]. Non-Hodgkin lymphomas (NHLs) are the most common adult hematological malignancies, followed by acute myeloid leukemia (AML), plasma cell diseases, chronic myeloid leukemia (CML), and acute lymphoblastic leukemia (ALL). The median age for NHL, AML, and myeloma is 65, 68, and 69 years, respectively. CML and ALL, on the other side, are generally seen in people in their 50s. In studies evaluating subtypes, it has been shown that the incidence of hematological malignancies increases with age, except for ALL and Hodgkin lymphoma (HL) [4].

Geriatric evaluation, performance position, presence, and number of comorbidities are important in hematological malignancies occurring in elderly patients. However, the characteristics of the tumor itself are limited to the histological structure [5]. The classification groups, including those specified by the European Leukemia Net (ELN) and the WHO, use prognostic classifications for leukemia based on traditional chromosomal re-arrangements combined with somatic gene mutations [6,7]. Despite all these advances, studies on tumor genomic structure are limited. In addition, hematological malignancies occurring in elderly patients progress differently from younger patients and show different genetic structures. Today, further investigations with New Generation Sequencing (NGS), in addition to cytogenetic data, may provide more detailed information [6]. For these reasons, in this retrospective study, it was aimed to determine the somatic gene variant profiles and gene fusion profiles of elderly and young acute leukemia patients to reveal the existing genetic differences and to compare the results.

## 2. Materials and Methods

### 2.1. Ethics and Subjects

The study was approved by the Aydin Adnan Menderes University, Non-Interventional Clinical Research Ethics Committee (#2023/80). The Helsinki Declaration criteria were taken into consideration in the study.

Adult patients aged 18 years and over who were referred to the Molecular Pathology Laboratory from the Hematology Clinic of the Aydin Adnan Menderes University, Faculty of Medicine Hospital, between January 2018 and December 2022, were screened retrospectively, and 204 cases with a diagnosis of acute leukemia were included in the study. Patients whose diagnosis were not confirmed were excluded from the study. Age, gender, and diagnostic subgroup of the patients at the time of diagnosis were obtained through the electronic database for patient follow-up in the Molecular Pathology laboratory. The data obtained were grouped according to age and diagnostic subgroups for NGS panel and Real-Time PCR fusion gene analyses. Accordingly, patients aged 18–64 were determined as the young group, while patients aged 65 and over were determined as the elderly group. Acute leukemias are grouped under separate subheadings, as AML and ALL.

### 2.2. DNA Isolation

Genomic DNA isolation from the bone marrow materials was performed with the QIAamp Blood Mini Kit (Qiagen, Hilden, Germany). DNA quantification and purity were measured with a Qubit dsDNA BR Assay system (Invitrogen, Carlsbad, CA, USA) using an Agilent 2100 Bioanalyzer (Agilent, Santa Clara, CA, USA).

### 2.3. RNA Isolation

RNA isolation from the bone marrow materials was performed using the QIAamp RNA Blood Mini Kit (Qiagen). RNA purity and amount were determined by the Qubit dsDNA BR Assay system (Invitrogen) and using the RNA Integrity Number (RIN) from the Agilent 2100 Bioanalyzer (Agilent), and samples suitable for these measurements were included in the study. The next steps were continued with a minimum of 0.15 μg total RNA and with a minimum RNA concentration of 8 ng/μL.

### 2.4. cDNA Synthesis and Fusion Gene Panel Assay

The chromosomal translocation/fusion gene panel was performed according to the manufacturer’s protocol using the HemaVision^®^-28Q Panel (DNA Diagnostic A/S Risskov, Aarhus, Denmark) (Table 1). Ready-to-use cDNA and qPCR master mixes were included in the kit. Purified RNA was added to the ready-to-use cDNA reaction mix for cDNA synthesis. As a control for the functionality of the qPCR reaction and the correct transfer of cDNA aliquots into the subsequent qPCR reaction, the internal amplification control (IAC) was included in the cDNA reaction mix. The resulting cDNA was added to 23 ready-to-use qPCR reaction tubes containing specific PCR primers and probes for detection of fusion genes, three reference genes, and an IAC. The qPCR reaction was performed on a Multiplex-Real-Time qPCR (Rotor-Gene 3000, Corbett Research, Qiagen) instrument with optical filters to detect FAM, ROX, and CY5 fluorescence. The translocation specific primers attached to exons in the fusion gene and enabled amplification of the region containing the breakpoint. The primers in the kit are designed to detect multiple clinically relevant breakpoints/splicing variants, thereby screening for 28 leukemia-associated chromosome translocations and more than 145 breakpoints. *B2M*, *GUS*, and *ABL* genes were selected as reference genes to control the integrity of the RNA sample and the functionality of both cDNA and qPCR reactions. A translocation positive control (HemaVision Q Positive Controls, HV05-PCQ) supplied by the manufacturer was used in the experiments.

### 2.5. NGS Human Myeloid Neoplasm Panel Test

In the study, the use of the Human Myeloid Neoplasm QIAseq Targeted DNA Panel was preferred as the NGS panel test. This panel encompasses 141 genes commonly mutated in myeloid neoplasms. It focuses on the most relevant variants in myeloid neoplasms by incorporating compiled databases such as Cancer Gene Census and Catalogue of Somatic Mutations in Cancer (COSMIC), as well as scientific networks including the Cancer Genome Atlas (TCGA) and the latest whole genome/exome sequencing studies [8]. An NGS panel test (Myeloid Neoplasms Panel, DHS-003Z, Qiagen, Hilden, Germany) (Table 2) was performed following the manufacturer’s protocol. Following DNA isolation, the NGS workflow continued with target enrichment, library preparation, template preparation, sequencing, variant calling, variant classification, and interpretation. Quality control of the prepared libraries was performed with the Qubit dsDNA BR Assay system (Invitrogen, Carlsbad, CA, USA). Sequencing was performed using a MiniSEQ High Output Reagent Cartridge (Illumina, Inc., San Diego, CA, USA) on a MiniSEQ NGS platform (MiniSEQ, MN00676, Illumina, Singapore).

### 2.6. NGS Variant Assessment

The variants were analyzed with pathological and clinical findings as well as with the automatic bioinformatics support of Qiagen Clinical Insight Interpret 8.1.202021. Variants were classified into four tiers (Tiers I–IV) based on cancer diagnosis, prognosis, and/or relevance to therapeutics. The variants of strong clinical significance were classified as Tiers I and II. The variants of unknown clinical significance due to insufficient evidence were classified as Tier III, and the variants with sufficient evidence to be classified as benign or possibly benign were classified as Tier IV.

### 2.7. Statistical Analysis

The Statistical Package for Social Sciences (SPSS) (Chicago, IL, USA) for Windows 22.0 program was used for statistical analysis. Chi-squared analysis was used to compare categorical data. Data are shown as numbers and percentages. A level of *p* < 0.05 was considered statistically significant.

## 3. Results

### 3.1. Clinical Findings

A total of 204 cases (69 female/135 male) were included in the analyses. When all cases were evaluated together, the most frequently diagnosed acute leukemia was found as AML (n = 175), representing 85.8% of the cases, followed by ALL (n = 29), constituting 14.2% of the cases.

When grouped by age, 56.8% of the cases were 65+ years old (n = 115). Accordingly, elderly cases were most frequently diagnosed with AML (n = 95, 82.6%) and ALL (n = 20, 17.4). It was found that 43.2% (n = 89) were young patients aged 18–64. The most frequently diagnosed acute leukemia in this age group was AML (n = 80, 89.9%) and ALL (n = 9, 10.1%). There was no statistically significant difference between groups (*p* = 0.09) in the distribution of acute leukemia.

### 3.2. Molecular Findings

#### 3.2.1. Gene Fusions

The most common gene fusions detected in AML cases in the young group are *RUNX1::RUNX1T1* (10%), *CBFB::MYH11* (10%), and *PML::RARA* (6.3%). These fusions are followed by *DEK::NUP214* (1.25%), *MLL::MLLT4* (1.25%), *MLL::MLLT3* (1.25%), *MLL::MLLT10* (1.25%), and *BCR::ABL1* (1.25%) fusions. In ALL patients, *BCR::ABL1*, *MLL::AFF1*, *SET::NUP214*, *STIL::TAL1*, *MLL::MLLT10*, and *MLL::ELL* fusions were each detected in 11.1% of the cases.

Gene fusions observed in AML cases in the elderly group are *RUNX1::RUNX1T1* (10.52%), *CBFB::MYH11* (9.47%), *PML::RARA* (7.36%), *SET::NUP214* (3.2%), *STIL::TAL1* (3.2%), *MLL::AFF1* (3.2%), *MLL::MLLT10* (3.2%), and *BCR::ABL1* (1.25%). The most common fusion detected in CML cases was *BCR::ABL1* (93%). The *MLL::AFF1*, *SET::NUP214*, *STIL::TAL1*, and *MLL::MLLT10* fusions were each detected in 25% of ALL patients. In addition, *BCR::ABL1* fusion was detected in 20% of ALL patients. All gene fusions in both groups are shown in Table 3.

#### 3.2.2. Somatic Variants

**Figure 1:**
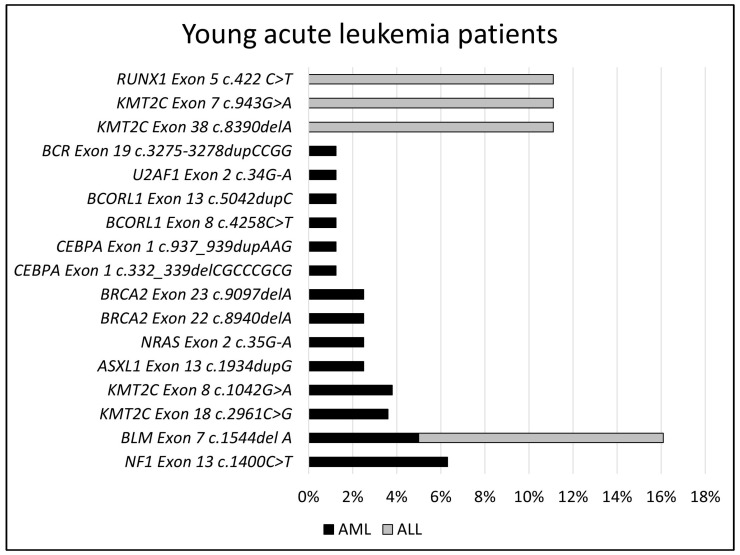
Pathogenic somatic gene variation distributions in young group.

When the groups were evaluated in terms of pathological somatic gene variants, the most common pathogenic gene variants in AML patients in the young group were *NF1* (6.3%), *BLM* (5%), *KMT2C (Exon18 c.2961C>G* (3.6%), and *Exon8 c.1042G>A* (3.8%)) variants. These pathogenic gene variants were followed by *ASXL1* (2.5%), *NRAS* (2.5%), *BRCA2 (Exon22 c.8940delA* (2.5%) and *Exon23 c.9097delA* (2.5%)), and each at 1.25% of *CEBPA (Exon1 c.332_339delCGCCCGCG* and *c.937_939dupAAG)*, *BCORL1 (Exon8 c.4258C>T* and *Exon13 c.5042dupC)*, *U2AF1*, and *BCR* pathogenic gene variants. The three most common possible pathogenic gene variants in AML patients in this group are *NF1* (5%) and *KMT2C (Exon18 c.2917A>G* (3.75%) and *Exon15 c.2578C>T* (3.75%)). The most common pathogenic gene variants in ALL patients are *BLM*, *KMT2C (Exon38 c.8390delA* and *Exon7 c.943G>A)*, and *RUNX1*, each present at 11.1%. The variants of *KMT2C (Exon18 c.2917A>G* (5%) and *c.2961A>G* (5%)) and *BCR* (5%) were detected as possible pathogenic gene variants. Figure 1 shows the pathogenic somatic gene variants observed in the young group.

**Figure 2:**
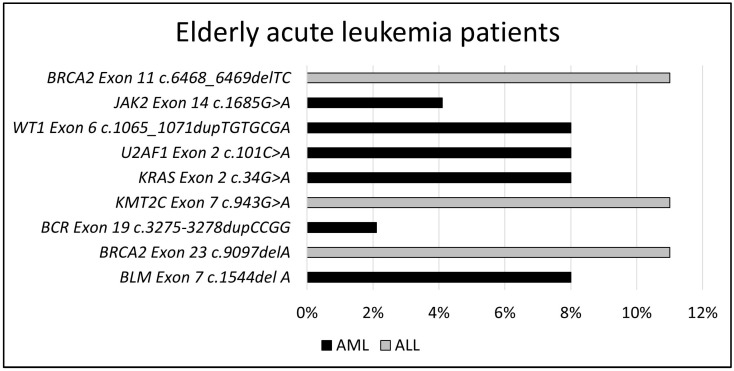
Pathogenic somatic gene variation distributions in elderly group.

The most common pathogenic gene variants in elderly AML patients are *BLM* (8%), *KRAS* (8%), *U2AF1* (8%), *WT1* (8%), *JAK2* (4.1%), and *BCR Exon19 c.3275-3278dupCCGG* (2.1%). The *BCR Exon 18 3143_3146dupCCGG* (2.1%) variant was identified as a possible pathogenic gene variant in these cases. The most common pathogenic gene variants in ALL patients are *BRCA2 (Exon11 c.6468_6469delTC* (11%) and *Exon23 c.907delA* (11%)) and *KMT2C* (*Exon14 c.2468delTinsAC* (11%) and *Exon7 c.943G>A* (11%)). In these cases, the possible pathogenic gene variant is *KMT2C* (11%). Figure 2 shows the pathogenic somatic gene variants observed in the elderly group.

## 4. Discussion

The disparities in leukemia occurrence across regions may be elucidated by variations in the quality and accessibility of healthcare systems. In our study, 69 (31.9%) of 204 patients with acute leukemia were female and the remaining 135 (68.1%) were male, and the M/F ratio was approximately 1.95. While factors such as gene/environment interactions likely contribute to the etiology, our study observed a consistent pattern, reflecting global trends where leukemia rates in males surpass those in females, maintaining a consistent ratio of 1:4 [5]. In this context, the high male ratio in our acute leukemia cases is consistent with the scientific literature.

Changes occur in the body with age that support tumor growth and metastasis. Studies on the subject show that cancer onset in older adults is much easier than in young people [9]. In this study, elderly patients constituted 56.8% of the subjects, consistent with the literature.

The most common leukemia observed in the elderly patient population is AML. The prevalence of AML in geriatric people is 17.9/100,000 [10]. In the current study, AML was the type of acute leukemia for which we found the most cases, and its incidence increased in the elderly.

In the elderly patient population, the approach to AML patients is of great importance. Patients with AML are treated by dividing them into risk groups according to WHO and ELN. In both guidelines, no distinction was made for elderly patients [6,7]. Groups have used prognostic classifications for AML, based on traditional chromosomal rearrangements combined with somatic mutations. In the tumor tissue of AML patients, the presence of *RUNX1::RUNX1T1* and *CBFB::MYH11* fusions positively affects the course of the disease. However, *DEK::NUP214*, *KMT2A (MLL)*, *BCR::ABL*, *GATA2*, *MECOM (EVI1)*, monosomy 5 or deletion 5q, monosomy 7, monosomy 17/abn (17p), complex karyotype, and monosomal karyotype have negative effects on the course of AML disease [6]. In the current study, the most common fusions in young AML patients were *RUNX1::RUNX1T1*, *CBFB::MYH11*, and *PML::RARA*, consistent with the WHO classification. These fusions were followed by *MLL::MLLT4*, *DEK::NUP214*, *MLL::MLLT3*, *MLL::MLLT10*, and *BCR::ABL1* fusions, similar to the literature. Again in the current study, the most common fusions in AML cases in the elderly group were *RUNX1::RUNX1T1*, *CBFB::MYH11*, *PML::RARA*, *BCR::ABL1*, *SET::NUP214*, *STIL::TAL1*, *MLL::AFF1*, and *MLL::MLLT10*. Gene fusions detected in both groups were similar, but gene fusions were observed in more patients in the elderly group. These results were in line with the WHO classification [6]. The *BCR::ABL1* fusion detected in AML patients in both groups was characterized by the presence of bone marrow and peripheral blood myeloblasts, with features ranging from minimal differentiation to granulocytic maturation, a rare type of AML (≤1%) with recurrent genetic abnormalities, and was associated with poor prognosis [11]. However, this fusion was seen more frequently (1.25%) in our study than in the literature in both groups. This may be due to the small number of AML patients.

With a literature-similar profile [12], 22.2% of young ALL patients had *BCR::ABL1*, *SET::NUP214*, *STIL::TAL1*, *MLL::AFF1*, and *MLL::MLLT10* fusions, and 11.1% had *MLL::ELL* fusion. Similar fusions, except for the *MLL::MLLT1*, were also detected in the elderly group at a rate of 25%. In addition, *BCR::ABL1* fusion was detected in 20% of ALL patients. 25% of young CLL patients had *SET::NUP214*, *STIL::TAL1*, *MLL::AFF1*, and *MLL::MLLT10* fusions, while no fusions were observed in CLL patients in the elderly group.

In most myeloproliferative neoplasms, one or more somatic mutations are present that activate a signaling pathway, providing a proliferative advantage to neoplastic cells. Myeloid neoplasms are known to have chromosomal translocations that regulate *PML-RARA*, *CBFB-MYH11*, and *RUNX1-RUNX1T1* myeloid transcription factors and *JAK2*, *FLT3*, *KIT*, *and RAS*, activating driver mutations in signaling pathways. In addition, mutations in the *CEBPA*, *RUNX1*, *CBFB*, and *NPM1* myeloid transcription factors, in the *SF3B1*, *U2AF1*, *SRSF2*, and *ZRSR2* splicing machinery components, in the *ASXL1*, *BCORL1*, *EZH2*, *DMNT3A*, *TET2*, *IDH1*, and *IDH2* epigenetic regulators, in the *STAG2*, *SMC1A*, *SMC3*, and *RAD21* cohesion complex, and in the *TP53*, *WT1*, and *PHF6* tumor suppressors are also commonly present in myeloid neoplasms [8,13,14,15]. The presence of a driver mutation is a diagnostic criterion for most myeloproliferative neoplasms according to the WHO classification. On the other hand, the presence of driver-independent mutations may have prognostic significance, as it can serve as presumptive evidence for clonality. In myeloproliferative neoplasms, the potential benefits of detecting somatic gene variations include identifying the *JAK2*, *CALR*, and *MPL* driver mutations, detecting other genes such as *ASXL1* that could provide prognostic information, aligning with targeted treatment options (e.g., *TP53* and *MDM2* inhibitors), and/or improving diagnostic categorization (e.g., *SF3B1*) [8,15]. Therefore, this retrospective study presents the results of NGS-based somatic gene profiles in both groups (young and elderly) of acute leukemia patients.

In molecular evaluation, *NPM1* mutation without *FLT3-ITD* or low expression of *FLT3-ITD* and biallelic mutant *CEBPA* have a positive effect on the AML. In both WHO and ELN classifications, certain somatic mutations (*ASXL1*, *BCOR*, *EZH2*, *SF3B1*, *SRSF2*, *STAG2*, *U2AF1*, and *ZRSR*), together with chromosomal rearrangements, have taken their place in the treatment approach as well as diagnosis [6,7]. In the study of Duncavage et al., genes required for diagnosis and risk stratification in AML were indicated as *ASXL1*, *BCOR*, *CEBPA*, *DDX41*, *EZH2*, *FLT3-ITD*, *FLT3*, *TKD*, *IDH1*, *IDH2*, *NPM1*, *RUNX1*, *SF3B1*, *SRSF2*, *STAG2*, *TP53*, *U2AF1*, and *ZRSR2*. The genes that are recommended to be tested at the time of diagnosis and used in the follow-up of the disease are designated as *ANKRD26*, *BCORL1*, *BRAF*, *CBL*, *CSF3R*, *DNMT3A*, *ETV6*, *GATA2*, *JAK2*, *KIT*, *KRAS*, *NRAS*, *NF1*, *PHF6*, *PPM1D*, *PTPN11*, *RAD21*, *SETBP1*, *TET2*, and *WT1* [16]. In the current study, *ASXL1* variants detected in young AML patients and *U2AF1* variants detected in elderly AML patients fit the WHO classification. In this study, *CEBPA*, *RUNX1*, *BCORL1*, and *NF1* variants detected in young AML patients and *WT1*, *KRAS*, and *JAK2* variants detected in elderly AML patients were consistent with the literature in diagnosis and risk stratification and disease follow-up.

*BLM* mutations are associated with extreme cancer susceptibility in AML patients in both groups and in ALL patients in the young group. In their case-control study, Broberg et al. investigated 26 tagged single nucleotide polymorphisms (tagSNPs) in *RMI1*, *TOP3A*, and *BLM* and their associations with AML/myelodysplastic syndromes, malignant melanoma cancer, breast cancer, and bladder cancer risk. They reported that *BLM* polymorphisms increased the risk of breast cancer [17]. It has also been reported in the literature that ALL and treatment-related AML developed in a girl with Bloom syndrome. The girl was previously reported to have a *BLM c.3415C>T* nonsense mutation, and a newly formed *BLM frameshift c.1624delG* mutation developed [18]. In this sense, there are insufficient studies supporting the role of *BLM* mutations in the development of AML. The detection of *BLM* mutations in both study groups can be attributed to the high sensitivity of NGS or the fact that it was a study based on an ethnically different population.

Recent genome-wide studies of cataloging somatic gene variations in cancer have identified mutations in intergenic sequences encoding regulatory elements and in *KTM2C (MLL3)* and *MLL4* in both hematological malignancies and solid tumors. It has been reported that the *KMT2C* and *MLL4* genes are frequently mutated in many different forms of cancer, some of which include bladder cancer, breast cancer, colon cancer, gastric cancer, liver cancer, medulloblastoma, and non-Hodgkin lymphoma [16]. However, *KMT2C* somatic gene mutations have not been identified in hematological malignancies, except for NHL [16]. In the current study, pathogenic and possible pathogenic somatic gene variants in different exons of *KMT2C* were detected in all acute leukemia groups in the young group and in ALL patients in the elderly group. This can be attributed to the recent addition of NGS data to the literature and to the fact that leukemia is a disease group with a heterogeneous genetic profile and different ethnic origins of patients.

*BRCA* mutations are associated with an increased risk of breast/ovarian cancers and other solid tumors [19,20]. There are few reports indicating an association between *BRCA* mutations and hematological malignancies [20,21]. In a study by Yin et al., they presented a new *BRCA2* mutation, *c.8434_8435insTT*, associated with multiple hematological malignancies and solid tumors in a single family [20]. In our study, *BRCA2* somatic gene variation was detected in elderly ALL patients and young AML patients. In this sense, the relationship between *BRCA2* mutation and hematological malignancies is remarkable.

As a result, acute leukemias can occur at any age, but the incidence of cancer increases with age and is more common in elderly patients. When the current study was evaluated in general, acute leukemias were mostly detected in the elderly group, in line with the literature. The most common type of acute leukemia in both young and elderly groups was AML. Both groups had similar fusion gene profiles, but the fusion load was higher in the elderly group. When we evaluated the groups in terms of somatic gene variations, there was not much similarity between the groups, and the variation load was higher in the elderly group. Considering the different somatic variation profile, it is understood that the genetic structure of tumor cells is different. The difference in tumor genetics in these patients may be an indicator of disease course and aggression. The frequent detection of *BLM* and *KMT2C* variations in our patients suggested that these variations may have prognostic significance in acute leukemia. The fusion profile was not similar to the somatic variation profile in the patients. This indicated the need to validate the fusion profile with NGS-based fusion gene panels or NGS RNA Seq data.

## 5. Conclusions

In this sense, these results are the first data reported from Türkiye. As a result, it is important to evaluate each of the gene variants occurring in acute leukemias separately and to know their distribution in elderly patients for the development of health policies. Understanding the genetic structure of tumors in elderly patients may lead to better treatment determinations. In conclusion, revealing the distribution of leukemia in elderly patients has an important place in the development of health policies.

## 6. Limitations

Patients with uncertain diagnosis were not included in the study due to the exclusion criteria. Therefore, the inclusion of relatively few leukemia cases in the study can be stated as a limitation. Study data can be validated with larger patient numbers, and fusion gene results with NGS-based fusion gene panels or NGS RNA Seq data.

## Figures and Tables

**Table 1:** Chromosomal translocations/gene fusions—HemaVision^®^-28Q Panel.

Translocation	Fusion Gene	Translocation	Fusion Gene
*t(15;17)(q24;q21)*	*PML::RARA (bcr2, V)*	*del(1)(p32)*	*STIL::TAL1*
*inv(16)(p13;q22)*	*CBFB::MYH11*	*t(9;22)(q34;q11)*	*BCR::ABL1 (m-bcr, P190)*
*t(8;21)(q22;q22)*	*RUNX1::RUNX1T1*	*t(9;9)(q34;q34)*	*SET::NUP214*
*t(15;17)(q24;q21)*	*PML::RARA (bcr1, L)*	*t(9;22)(q34;q11)*	*BCR::ABL1 (M-bcr, P210)*
*t(9;11)(p22;q23)*	*MLL::MLLT3*	*t(9;22)(q34;q11)*	*BCR::ABL1 (μ-bcr, P230)*
*t(15;17)(q24;q21)*	*PML::RARA (bcr3, S)*	*t(11;17)(q23;q21)*	*ZBTB16::RARA*
*t(11;19)(q23;p13.3)*	*MLL::ELL*	*t(9;12)(q34;p13)*	*ETV6::ABL1*
*t(16;21)(p11;q22)*	*FUS::ERG*	*t(5;12)(q33;p13)*	*ETV6::PDGFRB*
*t(12;22)(p13;q11::12)*	*ETV6::MN1*	*t(10;11)(p12;q23)*	*MLL::MLLT10*
*t(6;9)(p23;q34)*	*DEK::NUP214*	*t(1;11)(q21;q23)*	*MLL::MLLT11*
*t(1;11)(p32;q23)*	*MLL::EPS15*	*t(X;11)(q13;q23)*	*MLL::FOXO4*
*t(6;11)(q27;q23)*	*MLL::MLLT4*	*t(11;17)(q23;q21)*	*MLL::MLLT6*
*t(1;19)(q23;p13)*	*TCF3::PBX1*	*t(3;21)(q26;q22)*	*RUNX1::MECOM*
*t(12;21)(p13;q22)*	*ETV6::RUNX1*	*t(5;17)(q35;q21)*	*NPM1::RARA*
*t(11;19)(q23;p13.3)*	*MLL::MLLT1*	*t(3;5)(q25.1;q35)*	*NPM1::MLF1*
*t(4;11)(q21;q23)*	*MLL::AFF1*	*t(17;19)(q22;p13)*	*TCF3::HLF*

**Table 2:** Myeloid neoplasm NGS gene panel.

*JAK1*, *JAK2*, *JAK3*, *ADA*, *ANKRD26*, *ATM*, *ATRX*, *BCL6*, *BCOR*, *BCORL1*, *BCR*, *BIRC3*, *BLM*, *BRCA1*, *BRCA2*, *C17orf97*, *CARD11*, *CBLCBLC*, *CDKN2A*, *CEBPA*, *CHEK2*, *CREBBP*, *CRLF2*, *CSF1R*, *CSF3R*, *CTCF*, *CUX1*, *DAXX*, *EGFR*, *FLT3*, *ELANE*, *EP300*, *ETV6*, *EZH2*, *FAM154B*, *FAM47A*, *FAM5C*, *FAS*, *FBXW7*, *FLRT2*, *GATA1*, *GATA2*, *GJB3*, *GNAS*, *HNRNPK*, *HRAS*, *IDH1*, *IDH2*, *IKZF1*, *IKZF3*, *KAT6A*, *KCNA4*, *KCNK13*, *KDM6A*, *KDR*, *KIT*, *LUC7L2*, *BRAF*, *MAP2K1*, *MLH1*, *MPL*, *MSH6*, *MYC*, *MYD88*, *NBN*, *NF1*, *NOTCH1*, *NPAT*, *NPM1*, *NRAS*, *NSD1*, *NTRK3*, *OR13H1*, *OR8B12*, *P2RY2*, *PAX5*, *PCDHB1*, *PDGFRA*, *PHF6*, *PML*, *PRAMEF2*, *RAD21 CARL*, *RB1*, *RELN*, *RUNX1*, *SETBP1*, *SF1*, *SF3A1*, *SF3B1*, *SH2B3*, *SH2D1A*, *SMARCB1*, *SMC1A*, *SMC3*, *SRSF2*, *STAG2*, *STAT3*, *STXBP2*, *SUZ12*, *TAL1*, *TERC*, *TERT*, *TET2*, *TNFRSF13B*, *TP53*, *TPMT*, *TUBA3C*, *XPO1 ASXL1*, *ASXL2*, *TSC1*, *TSC2*, *U2AF1*, *U2AF2*, *WAS*, *WRN*, *WT1*, *PRF1*, *PRPF40B*, *PRPF8*, *PTEN*, *PTPN11*, *KLHDC8B*, *KLHL6*, *KMT2A*, *KRAS*, *LRRC4*, *DDX41*, *DNM2*, *DNMT1*, *DNMT3A*, *EED*, *SRP72*, *PIK3R2*, *PIM1*, *PIM2*, *PIM3*, *PSMA1*

**Table 3:** Chromosomal translocations/fusion gene distributions between young and elderly groups with acute leukemia.

		*BCR::* *ABL1* (n/%)	*CBFB::* *MYH11* (n/%)	*PML::* *RARA* (n/%)	*RUNX1::* *RUNX1T1* (n/%)	*MLL::* *MLLT10* (n/%)	*MLL::* *MLLT3* (n/%)	*MLL::* *MLLT4* (n/%)	*DEK::* *NUP214* (n/%)	*MLL::* *MLLT1* (n/%)	Other Fusions (n/%)
**Young group** **(n = 89)**	**AML** **(n = 80)**	1/1.25	8/10	5/6.3	8/10	1/1.25	1/1.25	1/1.25	1/1.25	1/1.25	ND
	**ALL** **(n = 9)**	1/11.1	ND	ND	ND	ND	ND	ND	ND	1/11.1	1/11.1
**Elderly group ** **(n = 115)**	**AML** **(n = 95)**	2/1.25	9/9.47	7/7.36	10/10.52	ND	ND	ND	ND	ND	3/3.2
	**ALL** **(n = 20)**	4/20	ND	ND	ND	ND	ND	ND	ND	ND	5/25

ND: Not detected.

## Data Availability

Additional data are available upon request.
